# Mechanisms of Prenatal Programing: Identifying and Distinguishing the Impact of Steroid Hormones

**DOI:** 10.3389/fendo.2014.00052

**Published:** 2014-04-14

**Authors:** Thomas G. O’Connor, Emily S. Barrett

**Affiliations:** ^1^Department of Psychiatry, University of Rochester Medical Center, Rochester, NY, USA; ^2^Department of Obstetrics and Gynecology, University of Rochester Medical Center, Rochester, NY, USA

**Keywords:** developmental programing, sex steroids, prenatal maternal distress, child developmental outcomes, methodology

## Abstract

Developmental programing is gaining considerable leverage as a conceptual framework for understanding individual variability in human behavioral and somatic health. The current mini-review examines some of the key conceptual and methodological challenges for developmental programing research focused on fetal sex steroid exposure and physical, behavioral, physiological, and health outcomes. Specifically, we consider the bases for focusing on sex steroids, methods for assessing prenatal steroid hormone exposure, confounding factors, and the most relevant postnatal outcomes. We conclude with a brief consideration, based on current knowledge, of the applications of the existing findings for further research and practice.

The developmental origins of health and disease hypothesis and the programing mechanisms that underlie it are a major focus of current basic science and clinical health research. A common theme is that stress (or in humans, anxiety) experienced by the mother is transmitted to the fetus and the ensuing developmental changes may have long-lasting effects on offspring biology and behavior. The hypothalamic–pituitary–adrenal (HPA) axis – and particularly glucocorticoid exposure – has attracted attention as a likely mediating mechanism (1–3). However, it may be worthwhile to look beyond the HPA axis. In this mini-review, we consider the role that sex steroids may play in prenatal programing and identify strategies for managing some of the methodological challenges that have arisen.

## Programing Effects from Prenatal Stress/Anxiety: Sex Steroids

It is natural that HPA axis-mediated programing mechanisms have attracted substantial research attention given the dominant role of prenatal stress as a “risk phenotype” in the animal and, more recently, human research. The mediating role of glucocorticoids in programing fetal physiology may extend beyond prenatal psychosocial stress to also include exposure to other types of stressors, such as nutritional deprivation. Moreover, there are equally compelling reasons why an exclusive focus on a glucocorticoid-mediated mechanism will be inadequate and consideration of complementary mechanisms, notably sex steroid hormones, could be informative. For example, the HPA and hypothalamic–pituitary–gonadal (HPG) axes show considerable overlap and interaction (4); just as stress may affect sex steroid hormone production, so sex steroids may act on the stress response system.

One way of evaluating the likely importance of sex steroids in developmental programing is to examine sex differences in the associations between prenatal stress and outcomes in offspring. For instance, in animal models, male and female offsprings often show different patterns of developmental programing (5). In some cases, only one sex appears to be affected by prenatal stress (6), but in other research, the sexes show opposite directions of effect. For example, in a rat model, prenatal restraint stress was associated with increased anxiety in males but decreased anxiety in females compared to controls (7).

Whether or not there are sex differences in human studies of the effects of prenatal stress/anxiety on child outcomes is far from clear. Some examples have been reported for behavioral and cognitive development (8, 9) and the evidence for cardiovascular outcomes is strong (10). However, for several of the most widely researched stress-related outcomes, such as behavioral and emotional problems and HPA axis function, few, if any, robust and consistent sex differences have been found (11, 12). This inconsistency across human studies raises several critical issues. First, there is no reason to expect that responses to prenatal stress will differ by sex across all traits, so carefully selecting only those phenotypes of most relevance is important. Second, it is plausible that focusing on HPA axis-mediated mechanisms, to the exclusion of other mediators, may have limited our ability to detect and understand sex differences.

Two lines of study provide evidence of sex steroid involvement in stress-related developmental programing. In animal models, prenatal stress is associated with changes in anogenital distance (AGD), a marker of prenatal androgen exposure; these associations have now been noted in humans (13–15). Critically, the direction of effects differs by sex: prenatal stressed males show demasculinization and females show masculinization of AGD. Interestingly, in contrast to the literature on prenatal nutritional stress, in which males tend to be more affected postnatally (16), the effects of psychosocial stress on reproductive development appear to be stronger in females (13, 17). Although research on prenatal stress and reproductive health and development in humans is limited, the fact that AGD appears to be stable over time and has been linked to adult reproductive outcomes (18, 19) suggests the potential importance of this line of research. A related line of work suggests prenatal programing from sex steroids from testosterone in amniotic fluid on autistic behaviors (20, 21) as well as temperament and play behavior (9, 22) in childhood. These findings point to the need for further clinical research on developmental programing of sex steroids and their effects on human health and development.

## Methodological Challenges for Investigating Prenatal Programing Effects of Sex Steroids in Human Studies

There are several methodological challenges for future research on the possible programing role of prenatal sex steroid exposure; we consider several in this mini-review.

### Assessing fetal exposure

The first, most basic question is how to assess fetal exposure to sex steroid hormones. Several studies have inferred fetal exposure to sex steroids from prenatal maternal distress (13, 23), although direct evidence of an association has not been found, e.g., Ref. (24). Directly measuring fetal exposure to sex steroids remains a major challenge. In the past, many studies were able to measure hormones in amniotic fluid when there was clinical indication; however, amniocentesis is losing favor as a clinical diagnostic tool and is unlikely to be a viable measurement strategy moving forward. Less direct measures of fetal exposure may be obtained from the mother; saliva, serum, and hair have been suggested as potential candidates. Circulating maternal hormone levels may represent production by the fetus, placenta, and the mother herself, with the relative proportions differing by hormone and stage of gestation (25–27). Circulating maternal testosterone levels could be a useful index, but it is likely that most fetal androgens are aromatized by the placenta before reaching maternal circulation (28). Finally, if circulating maternal sex steroids are correlated with fetal exposure, it still remains unclear which maternal medium is most appropriate for measurement, given that there may be inconsistencies across media (29). In any event, there is good reason to suspect that peripheral maternal levels of sex steroids may have minimal influence on fetal exposure. One potentially valuable strategy to index fetal exposure is to examine placental gene expression (see below).

Indexing fetal exposure to sex steroids *in utero* is an essential but difficult task for research. The availability of non-intrusive and reliable estimates of fetal exposure has proved difficult and is a central challenge to overcome in future research.

### Distinguishing between correlated steroid hormone exposures

Distinguishing the impact of sex steroid hormones from other sources of programing is another major challenge for research. For example, cortisol and testosterone are positively correlated in fetal blood (30) and in amniotic fluid (31). Given that, it may be difficult to separate programing effects attributable to sex steroids from those attributable to glucocorticoids. One approach in past research is to examine natural experiments in which a medical condition, such as congenital adrenal hyperplasia (CAH), alters the fetal hormonal milieu in a well-characterized manner. Findings from studies of girls with CAH, for example, have helped to establish that prenatal exposure to sex steroids may program postnatal outcomes ranging from play behavior, to motor development, to personality (32–34). Interestingly, females with non-classical CAH have “female-typical” genitalia at birth (with evidence of impaired fertility later in life) (35), however, this does not rule out the possibility of subtly masculinized AGD, which would not be evident to a casual observer.

On the other hand, it is unclear if the findings can be generalized to non-clinical populations with fetal hormonal exposures within the normal range of variation. Other means of differentiating between glucocorticoid and sex steroid-based programing within healthy populations are needed. It is an interesting possibility that the developmental programing studies associated with maternal prenatal distress may have over-attributed effects to stress hormones because they have (largely) ignored sex steroids.

### Role of placental structure and function

A novel and potentially promising approach to indexing fetal steroid hormone exposure is to examine placental gene expression and epigenetic changes. As the main maternal–fetal interface, the placenta is of inarguable importance for understanding developmental programing. To date, research has been influenced by a glucocorticoid-mediated model. For instance, there has been great interest in the effects of stress on placental production of 11-β-hydroxysteroid dehydrogenase 2 (11BHSD2), an enzyme which shields the fetus from maternal cortisol by converting it to inactive cortisone (36). However, the placenta’s endocrine production and regulation clearly extend far beyond 11BHSD2; prenatal distress may affect other placental steroid hormone pathways, impacting sex steroidogenesis and activity. At the same time, remarkably few studies on developmental programing have looked forward from placental structure and function to clinical phenotypes in the offspring; exploratory work on this subject is needed.

There are obviously substantial hurdles to assessing placental gene expression; it is a methodology that poses significant collection, cost, and laboratory demands. In healthy pregnancies, moreover, we are limited to looking at placental morphology and physiology at birth rather than at critical periods earlier in gestation. Nevertheless, examining placental variation, particularly in steroidogenesis pathway activity, in relation to prenatal exposures and postnatal outcomes requires attention given the (other) inherent challenges of estimating fetal exposure to sex steroids in a non-intrusive, reliable way.

### Identifying relevant postnatal phenotypes

A final challenge to be considered is the selection of relevant postnatal phenotypes. Traits with notable sex differences may be the most fruitful starting points. Autism spectrum disorders and associated traits, for instance, differ quite notably in prevalence and presentation between the sexes (37). Play behavior is another strong candidate given the extensive evidence that from early childhood onward, males and females show clear preferences for sex-typical toys (38). Both autism and sexually dimorphic play behaviors have been associated with exposure to prenatal stress (17, 23), although further work is clearly needed. Other phenotypes that do not show consistent sex differences, such as temperament, may be less relevant to consider in this context.

Levels of circulating sex hormones are extremely low from shortly after birth until puberty. Nevertheless, even in infancy there appears to be sex differences in neurodevelopmental traits (39), suggesting that there may be prenatal, organizational effects of exposure of sex steroids. Other sex differences in development (in brain development, for instance), emerge later in childhood, but prior to the peripubertal increase in sex hormones and may plausibly be the product of *in utero* sex steroid programing. Studies showing sex differences in infancy are interesting because there are minimally detectable levels of sex hormones in circulation; that means that these infant sex differences may be induced by prenatal sex steroid exposure. There are other well-known differences between the sexes that are evident early in development, including physical growth and brain development, e.g., Ref. (40). A challenge for future research is to examine if these – and perhaps other – early-emerging sex differences in biology and behavior can be attributable in part to prenatal programing of sex steroid exposure.

## Conclusion

Research on human health and development is just beginning to translate the animal work on developmental programing effects of sex steroids. Further studies are needed to substantiate this emerging line of investigation and to provide a broader biological context in which to interpret the sizable research based on developmental programing associated with prenatal stress and HPA axis mechanisms, a literature which has begun to influence practice and policy, e.g., Ref. (41). Progress in the area of research will require the consideration and surmounting of several methodological challenges, which we have highlighted.

## Conflict of Interest Statement

The authors declare that the research was conducted in the absence of any commercial or financial relationships that could be construed as a potential conflict of interest.
